# Impact of Chinese palaeontology on evolutionary research

**DOI:** 10.1098/rstb.2021.0029

**Published:** 2022-03-28

**Authors:** Xiaoya Ma, Guangxu Wang, Min Wang

**Affiliations:** ^1^ Yunnan Key Laboratory for Palaeobiology, Institute of Palaeontology, Yunnan University, Chenggong Campus, Kunming 650504, People's Republic of China; ^2^ Centre for Ecology and Conservation, University of Exeter, Penryn Campus, Penryn, Cornwall TR10 9FE, UK; ^3^ State Key Laboratory of Palaeobiology and Stratigraphy, Nanjing Institute of Geology and Palaeontology, Chinese Academy of Sciences, Nanjing 210008, People's Republic of China; ^4^ Institute of Vertebrate Paleontology and Paleoanthropology, Chinese Academy of Sciences, Beijing 100044, People's Republic of China

**Keywords:** Chinese palaeontology, evolution, biota, origin, diversification

## 1. Background

Reference to fossils in Chinese literature can be traced back to the sixth century AD, but palaeontology as a modern scientific discipline was only established in China in the 1920s. ZHANG Hongzhao (1877–1951), DING Wenjiang (1887–1936), WENG Wenhao (1889–1971) and LI Siguang (1889–1971) are considered to be the founders of Chinese geology, with Ding playing a particularly significant role in the birth of Chinese palaeontology. In 1920, he invited US geologist Amadeus William Grabau (1870–1946) to be professor of palaeontology at the Department of Geology of Peking University and director of the Palaeontology Department at National Geological Survey of China, which marked the beginning of systematic Chinese palaeontological research. These early pioneers also educated the first generation of homegrown Chinese palaeontologists, including YANG Zhongjian (also known as CC Young), PEI Wenzhong, SUN Yunzhu and many more [1–3].

Despite difficult working conditions, the few pioneering Chinese palaeontologists made a great start, and in a short period of time Chinese palaeontology gained considerable international influence. Valuable publications include the first Chinese palaeobotanical paper by ZHOU Zanheng in 1923 [4], and the first Chinese invertebrate and vertebrate palaeontology monographs by SUN Yunzhu in 1924 [5] and YANG Zhongjian in 1927 [6], respectively. Some internationally influential fossil discoveries were also made around this period, such as the first fossil hominin skullcap discovered by PEI Wenzhong in 1929 during the excavations of the Peking Man Site at Zhoukoudian [2]. After the Second Sino-Japanese War started in 1937, many Chinese geologists and palaeontologists moved their work to Southwest China. In 1938, after BIAN Meinian discovered dinosaur fossils in Lufeng, Yunnan Province, Yang Zhongjian began to excavate and research the Lufeng dinosaur fauna, which became the birthplace of Chinese dinosaur research and one of the most abundant dinosaur faunas in the world.

Since the founding of New China in 1949, Chinese palaeontology has grown exponentially. For the first 30 years, Chinese palaeontology played a significant role in the applied fields of geological surveying, exploration (e.g. oil, coal and minerals) and education to meet the growing demands of a rapidly industrializing nation. During this period, many Chinese palaeontologists developed expertise in various groups of fossil organisms, which, along with the well-established biostratigraphy, provided a solid foundation for further palaeontological research in China.

Since the 1980s, Chinese palaeontology has achieved remarkable progress in fossil discoveries and scientific studies, having a profound impact on our understanding of evolutionary history. The springboard for these significant achievements is rooted in the rapid economic development in China, which provided substantial funding for scientific research, but also facilitated access to fossils through mining and construction activities (e.g. roads and property developments). Large-scale and long-term fossil excavations and collections were carried out by both professionals and amateurs, leading to many stunning discoveries. Increasing application of state-of-the-art techniques and international collaborations allowed Chinese palaeontologists to unlock the evolutionary significance of these fossils and greatly advanced palaeontological research.

In the light of recent progress and achievements, as well as to celebrate over one hundred years of Chinese palaeontological research, this issue selects some of the latest studies by the younger generation of Chinese palaeontologists to address how Chinese palaeontological research impacts our understanding of major evolutionary transitions.

## 2. Themes and topics

Chinese palaeontological research encompasses many episodes throughout the history of life on Earth and covers almost every aspect of palaeontological research. However, most internationally influential research focuses on some Lagerstätten (exceptionally well-preserved fossil assemblages) discovered in the past few decades, which significantly enriched our understanding of evolutionary history. This issue focuses on three key evolutionary transitions that have been considered research hotspots in China.

### (a) Origin and early diversification of multicellular organisms

For most of the 4.6-billion-years of Earth's history, the Earth was dominated by single-celled organisms until the Ediacaran period at 635–541 million years ago (Ma), when abundant, complex, multicellular organisms started to appear globally. However, the affinities and evolutionary implications of Ediacaran fossils have long been a subject of debate due to their bizarre body plans, and the fact that most Ediacaran organisms became extinct at the Ediacaran–Cambrian boundary at 541 Ma. Shortly after the boundary, all major animal groups suddenly appeared in the Cambrian fossil record, and this landmark evolutionary event is known as the ‘Cambrian Explosion’. Therefore, Ediacaran and Cambrian fossils are crucial for understanding key evolutionary events from the appearance of multicellular organisms to the early radiation of animal life, which remains one of the most challenging frontiers of palaeontological research. China has the most complete and fossiliferous strata covering the Ediacaran to Cambrian periods that we know of so far, including a dozen world-famous exceptionally well-preserved fossil biotas, which provide a unique window into this pivotal evolutionary transition.

**Figure 1.**
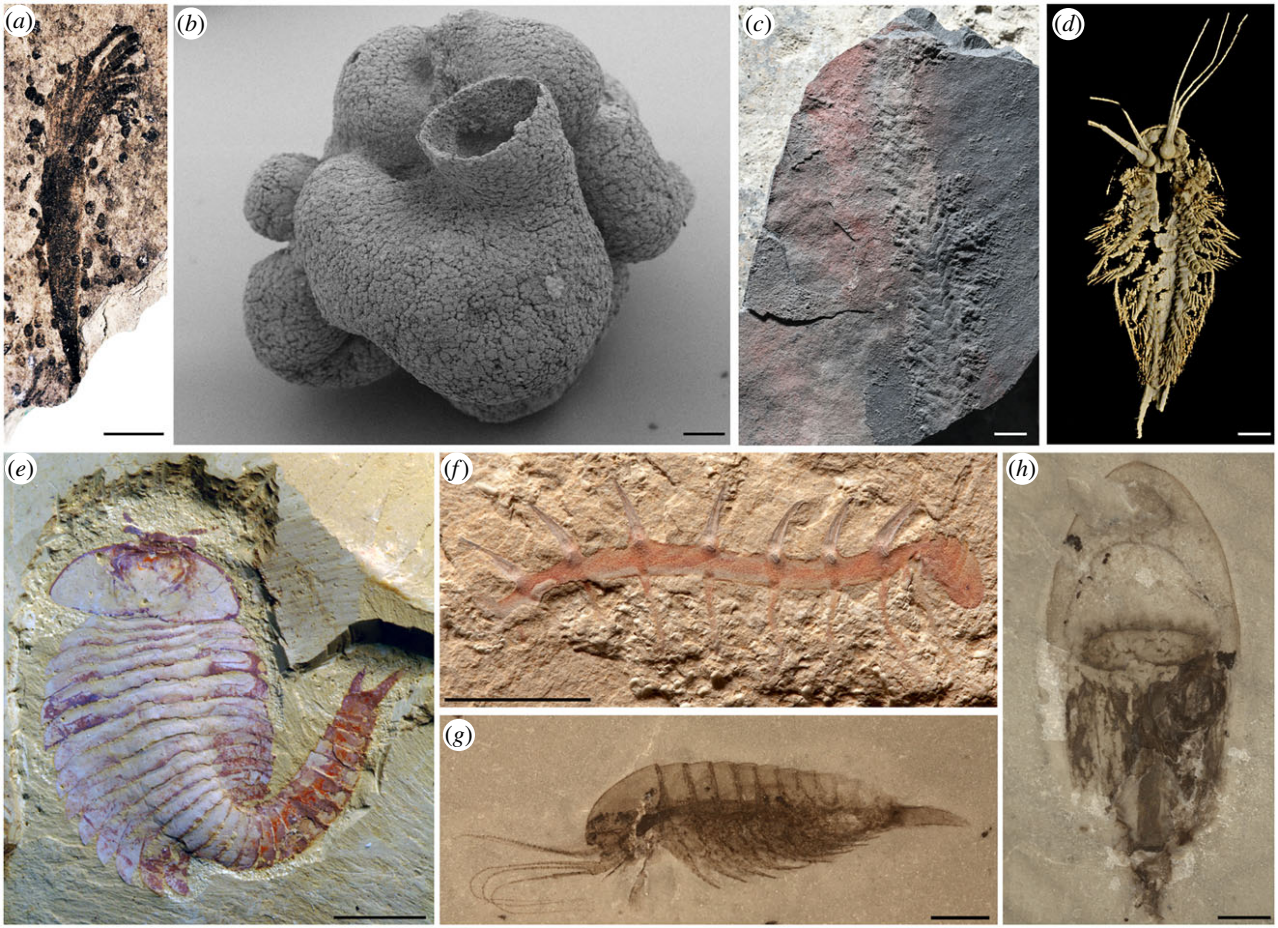
Selected early multicellular organisms from the Ediacaran and Cambrian biotas in South China. (*a*) *Lantianella laevis* from the early Ediacaran Lantian biota [7,8]*.* (*b*) *Eocyathispongia qiania* from the middle Ediacaran Weng'an biota [9]. (*c*) *Yilingia spiciformis* from the terminal Ediacaran Shibantan biota [10]. (*d*–*f*) Panarthropod fossils from the early Cambrian Chenjiang biota, (*d*) *Leanchoilia illecebrosa* [11], (*e*) *Fuxianhuia protensa* [12] and (*f*) *Hallucigenia fortis* [13]. (*g*,*h*) Specimens from the early Cambrian Qingjiang biota, (*g*) a medusoid cnidarian [14] and (*h*) *Leanchoilia* sp. [14]. Scale bars, 5 mm (*a*,*f*), 0.1 mm (*b*), 10 mm (*e*,*h*), 1 mm (*d*), 2 mm (*c*,*g*). Photo credits: (*a*), WAN Bin; (*b*), YIN Zongjun; (*c*), CHEN Zhe; (*d*), LIU Yu; (*e*,*f*), MA Xiaoya; (*g*,*h*), FU Dongjing.

The early Ediacaran Lantian biota (630–590 Ma) from Anhui Province, Southeast China is considered to be the earliest known fossil assemblage with macroscopic and morphologically complex organisms, some of which are suggested to be macroalgae and putative animals [7,8] (figure 1*a*). The middle Ediacaran Weng'an biota (*ca* 609–570 Ma) from Guizhou Province, South China is famous for three-dimensional phosphatized microfossils with unparalleled preservation to cellular and even subcellular fidelity. Many of these organisms have been interpreted as multicellular eukaryotes, including embryonic, larval and adult animals, although alternative interpretations have also been proposed [9,15,16] (figure 1*b*). The terminal Ediacaran Shibantan biota (551–543 Ma) in the Yangtze Gorges area of South China is one of the best-known examples of terminal Ediacaran fossil assemblages that unusually preserved macroalgae, biomineralizing tubular fossils, Ediacaran-type fossils, and abundant trace fossils in carbonate rocks. In recent years, some key findings of Shibantan trace fossils provide crucial insights into bilaterian behaviour and mobility across the Proterozoic–Phanerozoic transition [10,17,18] (figure 1*c*).

The Cambrian fossil record in China is unmatched in its abundance, continuity, diversity and preservation, with over 20 fossil assemblages discovered so far, furnishing robust evidence to enrich our understanding of the Cambrian Explosion. Immediately after the Ediacaran–Cambrian boundary, we see abundant Small Shelly Fossils (SSFs) at the base of Chinese Cambrian strata, such as the famous Meishucun fauna (*ca* 540–523 Ma), which is considered to mark the beginning of the Cambrian Explosion [19–21]. The Chengjiang biota (*ca* 518 Ma) is one of the oldest animal fossil assemblages that we yet know of, from which over 250 species across 18 phyla have been reported so far, providing a unique insight into the early radiation of animal life [13, and references therein] (figures 1*d*–*f*). The Chengjiang biota is famous for its exquisite soft-tissue preservation, even recording the oldest labile organ systems such as the nervous and cardiovascular systems [12,22]. The recently discovered Qingjiang biota (*ca* 518 Ma) with pristine carbonaceous preservation of organic features has produced a high number of new taxa that differ from those of the Chengjiang biota, demonstrating different Cambrian ecosystems across environmental gradients [14] (figure 1*g,h*). There are also other important Cambrian Lagerstätten in China, such as Kuanchuanpu [23], Xiaoshiba [24], Guanshan [25] and Kaili [26].

In this issue, we present four of the latest research articles on the origin and early diversification of multicellular organisms. The affinities of Ediacaran embryo-like fossils (EELFs) from the Weng'an biota remain highly debated, which hampered our understanding of their full evolutionary implications. To test competing hypotheses, Yin *et al*. [27] reconstructed a large number of Weng'an EELFs using submicron resolution X-ray tomographic microscopy, with a particular focus on their cell division pattern, associated developmental mechanisms and features of cell differentiation. The results demonstrate that the diversity and complexity of the developmental mechanisms of these EELFs are much higher than previously thought, and hence constrain their affinity as total-group metazoans.

After nearly four decades of research, new species of organisms are still being reported from the Chengjiang biota every year. Arthropods dominate this Cambrian marine community, comprising more than one-third of species. The huge diversity and exceptional preservation of these early arthropods provide crucial information on the origin and early diversification of this largest phylum. Fu *et al*. [28] report a new arthropod *Erratus sperare* gen. et sp. nov. from the Chengjiang biota, with unique trunk appendages composed of lateral anomalocaridid-type flaps and ventral subconical endopods. The authors suggest that these appendages represent an intermediate stage of biramous limb evolution, which is supported by their phylogenetic position. Therefore, this new species might represent the earliest occurrence of endopods within arthropods, shedding new light on the evolution of arthropod biramous appendages.

Research on arthropod appendages is important for understanding their phylogeny, evolutionary significance and functional ecology, but fossil arthropods' appendages are often buried in sediment. In recent years, the non-invasive micro-computed tomography (Micro-CT) technique has proved helpful in revealing Chengjiang arthropod appendages that were often pyritized and three-dimensionally preserved [11] (figure 1*d*). Schmidt *et al*. [29] use Micro-CT to reveal the ventral appendages of a rare arthropod *Pygmaclypeatus daziensis*, which shows an unexpectedly high degree of heteronomy. The animal's morphology indicates a nekto-benthic mode of life and a scavenging/detritus feeding strategy. The phylogenetic analysis suggests that a single exopodite lobe with paddle-like lamellae is ancestral for the clade of Artiopoda (including trilobites and their relatives).

The Cambrian Explosion and the Great Ordovician Biodiversification Event represent two major diversification events in animal evolutionary history, but they are characterized by strikingly different marine communities. Therefore, the Cambrian–Ordovician transition has long been a research topic of great interest. Shan *et al*. [30] explore the evolution of the palaeoecological patterns of the organic-walled phytoplankton (acritarchs) over the Cambrian and Ordovician using a selection of the most abundant taxa across China. The study demonstrates that early Cambrian acritarchs were relatively simple in structure, low in diversity and limited to inshore marine environments. However, from the late Cambrian to the Early Ordovician, acritarchs progressively extended to offshore habitats and then evolved to highly diverse assemblages with very complex morphologies. These results confirm the onset of the ‘Ordovician plankton revolution’.

**Figure 2.**
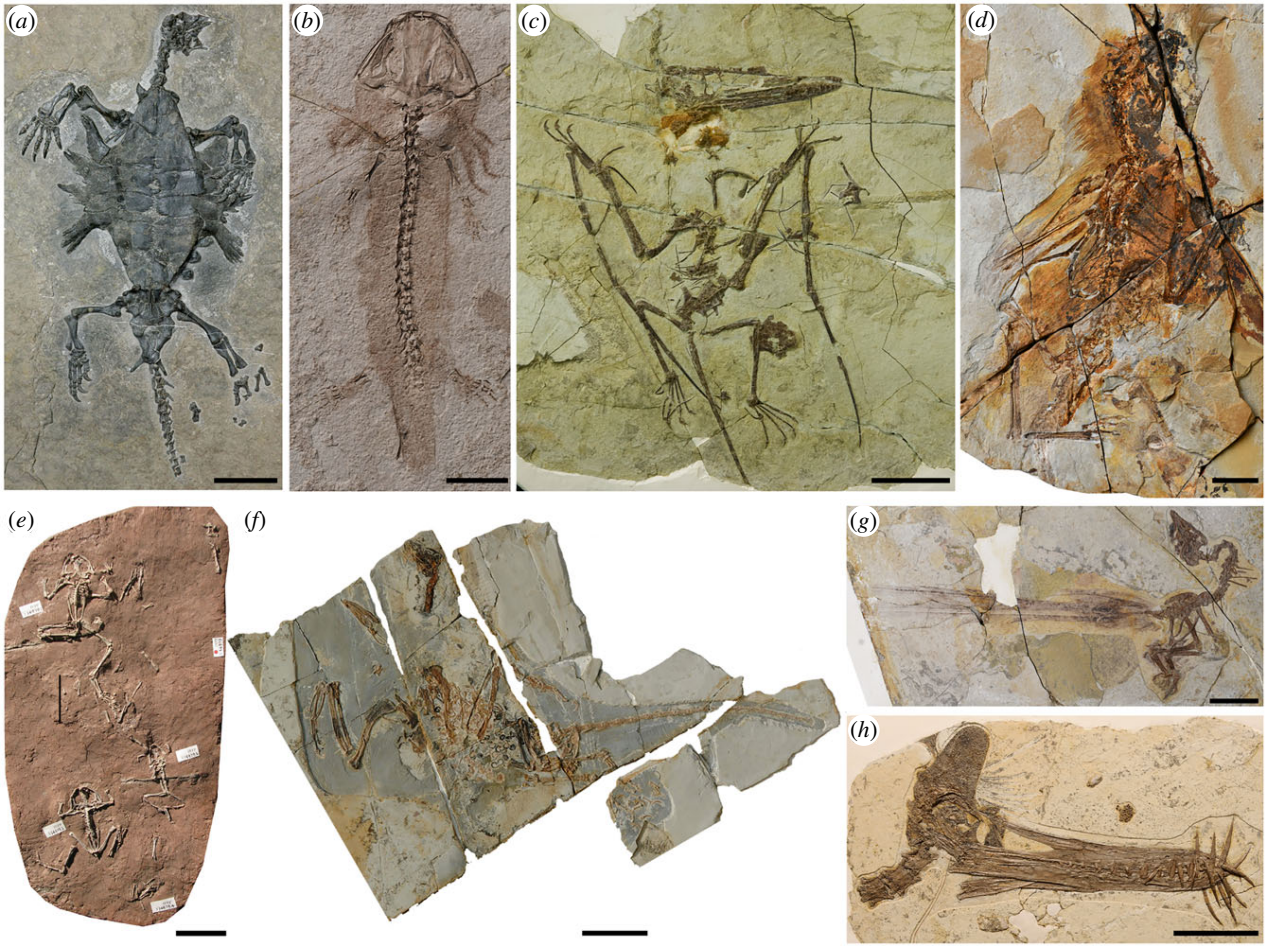
Selected vertebrate fossils from the Mesozoic lagerstätten in China. (*a*) A stem turtle *Odontochelys semitestacea* from the Middle Triassic Guanling Biota [35]. (*b*–*d*) Specimens from the Late Jurassic Yanliao Biota, (*b*) the salamander *Chunerpeton tianyiensis* [36], (*c*) the pterosaur *Darwinopterus linglongtaensis* [37] and (*d*) the scansoriopterygid theropod dinosaur *Ambopteryx longibrachium* [38]. (*e*–*h*) Specimens from the Early Cretaceous Jehol Biota, (*e*) four specimens of the frog *Liaobatrachus zhaoi* [39], (*f*) the holotype of the long-tailed bird *Jeholornis prima* [40], (*g*) the holotype of the enantiornithine bird *Yuanchuavis kompsosoura* [41] and (*h*) the holotype of the pterosaur *Guidraco venator* [42]. Scale bars, 5 cm (*a*,*c*,*e*–*g*), 2 cm (*b*,*d*), 10 cm (*h*). Photo credits: (*a*), LI Chun; (*b*,*h*), JIANG Shunxing; (*d*,*f*,*g*), WANG Min; (*c*,*e*), DONG Liping.

After the Ordovician, we see a significant rise of vertebrates in the marine ecosystem. China has nearly complete Palaeozoic to Mesozoic successions and some remarkable early vertebrate faunas. For example, exceptionally well-preserved late Silurian fishes from the Xiaoxiang Fauna of Qujing, Yunnan provided crucial information on the early diversification of jawed fishes [31–33]. The marine vertebrates across the Permo–Triassic transition are also well recorded in China, such as the Late Triassic Guanling biota of Guizhou, which is famous for its stunningly preserved marine reptiles, pelagic crinoids, rare fishes and ancestral turtles [34,35] (figure 2*a*).

### (b) Terrestrial organisms and ecosystems

After the origin and diversification of marine multicellular organisms, the third most profound evolutionary event is terrestrialization (the colonization of the land by marine plants and animals). The systematic study of fossil specimens of plants, insects and terrestrial vertebrates from the Late Palaeozoic to Mesozoic across China has revealed crucial information on the establishment and evolution of terrestrial organisms and ecosystems. Among these fossils, there are two world-renowned Lagerstätten, the Yanliao and Jehol biotas. Both biotas are famous for their remarkable preservation of a great diversity of fossil groups, including salamanders (figure 2*b*), pterosaurs (figure 2*c*,*h*), feathered dinosaurs (figure 2*d*), frogs (figure 2*e*), early birds (figure 2*f*,*g*), mammals, lizards, insects and plants. The Yanliao biota (166–159 Ma) is older than the Jehol biota (135–120 Ma), and, despite a superficial similarity, the two biotas differ significantly from each other in community composition. For example, pterosaur diversity is quite high in Yanliao biota, but by Jehol time, they have been usurped by early birds. Prior to the discovery of feathered dinosaurs from these Lagerstätten, there was no direct evidence of feathers occurring in dinosaurs, which provided crucial evidence that birds had evolved from theropod dinosaurs [36–45].

In this issue, we present five research articles that can be grouped within the theme of terrestrial organisms and ecosystems. A great diversification of early land plants occurred in the Devonian, as demonstrated by a series of globally distributed fossil floras. However, the spatial distribution and dispersal pattern of these plants remain enigmatic. The contribution by Wang *et al*. [46] records a new Early Devonian plant assemblage from Hezheng of Guizhou, Southwest China, from which two key forms (including the new zosterophyte *Pauthecophyton hezhangensis*) are systematically described. A review of regional flora data led the authors to recognize at least four biogeographic units, the Eastern Yunnan, Sichuan, Cathaysia and newly identified Guizhou sub-regions in South China during the Early Devonian. Also, they postulate that the Guizhou sub-region might have served as a land bridge between the Eastern Yunnan and Cathaysia sub-regions to facilitate plants' dispersal during this interval.

As the earliest vertebrates that conquered the sky, pterosaurs presumably played a central role in the terrestrial ecosystem of the Mesozoic Era and have long been represented in fiction as dragon/bat flying animals with a long rostrum packed with sharp teeth. Apart from their aerodynamically optimized skeletomuscular system, our knowledge about their key biological aspects, particularly the digestive system, has been impeded by ambiguous diet-related evidence. Jiang *et al*. [47] describe two specimens of the wukongopterid pterosaurs with associated gastric pellets from the Later Jurassic of China. These gastric pellets composed of fish bones not only lend further credence to the hypothesis that the wukongopterids were piscivorous, but also suggest that a two-chambered stomach, coupled with efficient antiperistalsis, long thought to be avian features, may have been in place in these pterosaurs.

The early evolution of mammals is one of the hottest topics in evolutionary biology, with conflicting results between palaeontological and molecular studies, and among palaeontological camps, regarding the divergence of major mammalian groups and interpretations of certain morphologies. The eutherians are the most speciose clade of living mammals, with their fossil record traced back to the Early Cretaceous. These early branching eutherians are central to our understanding of the characteristic mammalian features, such as the inner ear. Wang *et al*. [48] report a new species of eutherians from the 120-million-year-old deposits of the terrestrial Lagerstätte Jehol Biota. The new specimen shows a complete detachment of the middle ear from the lower jaw for the first time in eutherians, and also provides new data about cranial morphology and tooth replacement pattern of early eutherians.

Aposematism (whereby an organism advertises its dangerous nature to a potential predator by using bright colours or conspicuous markings) is one of the most prevalent antipredator strategies in the biological world, but a scarcity of fossil data hinders our understanding of its origin and early evolution. Xu *et al*. [49] report the discovery of aposematic coloration in *Monitelcana penalveri*, a new genus and species of the extinct orthopteran family Eclanoidea from mid-Cretaceous Kachin amber (approx. 99 million years old). The new species is the earliest known orthopteran preserved with aposematic coloration, offering unequivocal evidence that orthopterans had evolved aposematism by the Mid-Cretaceous. Together with diverse antipredator strategies previously documented, this find demonstrates that prey/predator interactions had been relatively complex in the Mid-Cretaceous forest ecosystem.

The monitor lizards (*Varanus* spp.), such as the Komodo Dragon, are one of the most successful clades of lizards with a wide geographical distribution across Asia, Africa and Australasia. However, little is known about the assembly of their characteristic body plan and biogeographic evolution due to the lack of well-preserved fossils. Dong *et al*. [50] report a new species of stem varanid from the Eocene of China, bridging the gap in the fossil record of monitor lizards. Furthermore, this study posits that the transition from Cretaceous varaniform lizards to *Varanus* took place in East Asia before dispersal to other regions.

### (c) Palaeoanthropology

Palaeoanthropology refers to the study of the origin and early evolution of humans. China is one of the few countries in the world particularly rich in early human fossils. This field in China began with the discovery of Peking Man at Zhoukoudian in the 1920s. In the past one hundred years, many more significant fossils have been found in various parts of the country, and the latest application of ancient DNA as well as radiocarbon dating has further revolutionized the study of human evolution in China [51,52].

Over the past few years, continuous discoveries of hominin fossils from the Middle Pleistocene of China have significantly advanced our knowledge of the tempo and pattern of the early evolution of hominins across eastern Eurasia. More importantly, these temporally well-constrained hominin fossils have challenged the previous hypothesis that *Homo erectus* in Asia underwent long periods of evolutionary stasis and ultimately went extinct in a so-called evolutionary backwater. In this issue, Liu *et al*. [53] performed morphometric analyses on those expanded hominin fossils from the Middle Pleistocene of China, which provides a testable model to explain the evolutionary process that underpins hominin morphological variations in eastern Eurasia from the early to the later Middle Pleistocene.

## 3. Looking forward

Chinese palaeontology has achieved remarkable progress in the past one hundred years, and there is much more that we can offer. Palaeontology is dependent on fossil material, and China has a nearly complete Palaeozoic to early Cenozoic geological record of both marine and terrestrial facies. Also, fossil exploration, excavation and collecting are continuing at a pace across the country, so we can expect more stunning discoveries of fossils and fossil sites in the future.

The development of biostratigraphy has made it possible to compare Phanerozoic strata with greater certainty. Among the 101 Global Stratotype Sections and Points (GSSP) identified by the International Commission on Stratigraphy (ICS), 11 GSSPs have been established in China, and more GSSP candidates are in extensive investigation and will be proposed for formal establishment. In the future, a high-resolution stratigraphic framework will be needed to solve some current controversies on stratigraphic correlation at regional, national and global levels, and to provide an accurate timeframe for palaeontological study.

In the new era of Chinese palaeontology, we will see increasing interdisciplinary approaches. In addition to traditional research methods, the younger generation of Chinese palaeontologists are increasingly applying the latest techniques (e.g. SEM EDX, Micro-CT, Synchrotron, X-ray fluorescence) to reveal the secrets of these ancient fossils. In recent years, Chinese palaeontologists have started to pay more attention to taphonomy to gain a better understanding of the process of fossilization and how it impacts our interpretations. Phylogenetic analysis is rapidly adopted by younger palaeontologists as a gold standard to investigate evolutionary relationships among different fossil groups and between fossil and extant taxa. With the development of evolutionary developmental biology and molecular phylogeny, combining fossil data with morphological and molecular data from extant species is becoming the most comprehensive method of assessing phylogenetic relationships and conducting time calibration on evolutionary events.

With the accumulation of fossil and stratigraphic data, the development of advanced analytical and modelling techniques, and increasing international collaboration, we are at the cusp of making breakthroughs to unravel macroevolutionary trajectories during important geological periods. To decipher how biotic evolution has influenced, and been influenced by the Earth's environment through geological history is one of the most challenging tasks in evolutionary research. For example, what are the causal factors for the major biodiversity radiation or mass extinction events? Through the increasing integration of multiple disciplines of palaeontology, palaeoecology, palaeogeography, biostratigraphy, geochemistry, sedimentology and palaeoclimatology, the study of the co-evolution of life and Earth may bear fruit in the new era of Chinese palaeontological research.

## Data accessibility

This article has no additional data.
